# Genome-wide analysis of DNA methylation during antagonism of DMOG to MnCl2-induced cytotoxicity in the mouse substantia nigra

**DOI:** 10.1038/srep28933

**Published:** 2016-07-06

**Authors:** Nannan Yang, Yang Wei, Tan Wang, Jifeng Guo, Qiying Sun, Yacen Hu, Xinxiang Yan, Xiongwei Zhu, Beisha Tang, Qian Xu

**Affiliations:** 1 Department of Neurology, Xiangya Hospital, Central South University, Changsha, 410008 Hunan, People’s Republic of China; 2 State Key Laboratory of Medical Genetics, Changsha, 410008 Hunan, People’s Republic of China; 3 Key Laboratory of Hunan Province in Neurodegenerative Disorders, Central South University, Changsha, 410008 Hunan, People’s Republic of China; 4 Neurodegenerative Disorders Research Centre, Central South University, Changsha, 410008 Hunan, People’s Republic of China; 5 Institute of Pathology, Case Western Reserve University, Cleveland, OH 44106. USA

## Abstract

Exposure to excessive manganese (Mn) causes manganism, a progressive neurodegenerative disorder similar to idiopathic Parkinson’s disease (IPD). The detailed mechanisms of Mn neurotoxicity in nerve cells, especially in dopaminergic neurons are not yet fully understood. Meanwhile, it is unknown whether there exists a potential antagonist or effective drug for treating neuron damage in manganism. In the present study, we report the discovery of an HIF prolyl-hydroxylase inhibitor, DMOG [N-(2-Methoxy-2-oxoacetyl) glycine methyl ester], that can partially inhibit manganese toxicity not only in the neuroblastoma cell line SH-SY5Y *in vitro* but also in a mouse model *in vivo*. A genome-wide methylation DNA analysis was performed using microarray hybridization. Intriguingly, DNA methylation in the promoter region of 226 genes was found to be regulated by MnCl2, while the methylation effects of MnCl2 could be restored with combinatorial DMOG treatment. Furthermore, we found that genes with converted promoter methylation during DMOG antagonism were associated across several categories of molecular function, including mitochondria integrity maintain, cell cycle and DNA damage response, and ion transportation. Collectively, our results serve as the basis of a mechanism analysis of neuron damage in manganism and may supply possible gene targets for clinical therapy.

Parkinson’s disease (PD) is a progressive disorder of the nervous system that mainly occurs in elderly individuals. Typical motor symptoms of PD include resting tremor, myotonia, slowing movements and peculiarities in gait and posture, while non-motor symptoms include cognitive impairments, disturbances of emotions and the mind, somatization disorders and autonomic nerve disorders^1^. Thus far, PD has typically been thought to be tightly correlated with ageing^2^, environmental^3^ and genetic factors^4^, nevertheless, the detailed mechanisms and pathogenesis of PD are not yet fully understood.

In the early 1980s, typical symptoms of PD were first found in drug addicts, when misusing MPTP (1-methyl-4-phenyl-1,2,3,6-tetrahydropyridine) in America^5^. At the same time, an extremely high incidence rate of PD was also found in humans in contact with agricultural chemicals, such as organophosphates, paraquat and insecticides, and these chemicals were thought to contain similar ingredients to MPTP^6^. PD has also been found to be associated with excessive manganese exposure^7^,^8^,^9^. Manganese is ubiquitous in the environment, mainly in the forms of inorganic salts or oxides, and it is widely used in industry, such as in iron and steel manufacturing, welding rods and dry battery manufacturing, etc. Although manganese is an essential nutrient metal required for the normal functioning of a variety of physiological processes, excessive exposure to manganese causes manganism, a progressive neurodegenerative disorder similar to idiopathic Parkinson’s disease (IPD), which is associated with neuronal injuries including a series of a variety of psychiatric and motor disturbances. Typical symptoms of manganism include postural instabilities, mood and psychiatric changes, and it is characterized by walking difficulties, dystonia, and kinesia, which are similar to those of Parkinson’s disease^10^,^11^. The detailed mechanisms of manganese-induced neurotoxicity to nerve cells, however, are still unclear.

Hypoxia-inducible factor 1 (HIF-1), composed of an HIF-1α and an HIF-1β subunit, is expressed in all metazoan organisms, and it functions as a transcription factor containing a basic helix-loop-helix-PAS domain^12^,^13^. In cells, HIF-1β is stably expressed, while protein levels of HIF-1α decrease quickly because of ubiquitination and 26S proteasome degradation in normal oxygen concentrations^14^. When oxygen concentrations are reduced (hypoxia), the normal degradation of HIF-1α is inhibited, leading to increased protein levels of HIF-1α and the formation of an HIF-1 protein dimer. This activated HIF-1 then recruits coactivator protein, binds the cis-response element together and participates in the transcriptional regulation of target gene^15^. The HIF-1 signalling pathway is involved in biological processes including angiogenesis and erythropoiesis^16^,^17^, which contribute to the promotion of and the increase in oxygen delivery to hypoxic regions. Moreover, HIF-1 also participates in the regulation of cell proliferation and survival, as well as glucose and iron metabolism^18^. In many human cancers, HIF-1 has been found to be overexpressed, consistent with its role in initiating angiogenesis and regulating cellular metabolism to overcome hypoxia. Recently, emerging results have shown that HIF-1 is also involved in impairments or improvements in neurodegenerative diseases including Parkinson’s disease, Alzheimer’s disease and amyotrophic lateral sclerosis^19^,^20^,^21^,^22^. Our previous results showed that the binding of HIF-1α to HRE (hypoxia response element) in the ATP13A2 gene activates its transcription and increases its expression dramatically. Meanwhile, overexpression of ATP13A2 antagonizes the cytotoxicity caused by excessive Mn exposure^23^. These results suggest that HIF-1 and its corresponding pathways might be potential therapeutic targets in various neurodegenerative diseases.

Epigenetics refer to heritable variations in functional genes and corresponding phenotypes that are not caused by changes in the DNA sequence. Epigenetic modulation is mainly comprised of DNA methylation, chromatin remodelling, histone modification, noncoding RNA (ncRNA) regulation etc. Of these, DNA methylation has attracted the most attention to date. In general, DNA methylation functions in normal development and is associated with a series of key biological processes including genome imprinting, X-Chromosome inactivation, the suppression of repetitive elements, etc. Increasing numbers of studies have shown that environmental exposure contributes to epigenetic modulation^24^,^25^. Thus, research into the epigenetics caused by environmental exposure will undoubtedly uncover the biological significance of environmental factors in diseases, including manganism or PD caused by excessive manganese exposure, and the regulations of genes expression caused by promoter methylation during the processes of corresponding therapies for manganism are also attractive.

In our current study, for the first time, we reveal the antagonistic effects of DMOG, an HIF-1 prolyl-hydroxylase inhibitor on the cytotoxicity caused by excessive manganese exposure not only in the human neuroblastoma cell line SH-SY5Y *in vitro* but also in the substantia nigra of the mouse brain *in vivo*.

The antagonistic effects of DMOG were first revealed in the dopaminergic neuron cell line SH-SY5Y, in which, decreased cell viability caused by MnCl2 treatment was partially restored by the administration of DMOG in a dose-dependent manner. Moreover, animal behavioural testing, including the accelerating rotarod test and the open field test, confirmed that over-dose manganese treatment greatly repressed the mouse action capability, consistent with a decreased tyrosine hydroxylase positive neurons number in mouse substantia nigra. A partial restoration of behavioural capability was observed when mice were treated with a combination of MnCl2 and DMOG in solution. Furthermore, using an Arraystar4 × 180 K microarray, we discovered a genome-wide methylation status change during the neurodegenerative disorder caused by excessive MnCl2 treatment and the antagonistic effect when we added additional DMOG. In this way, the variable DNA methylation status was profiled for the MnCl2-treatment groups, the combined MnCl2 and DMOG group and the mock controls. In the current study, for the first time, we reveal a global DNA methylation changes in DNA methylation caused by Mn exposure and their reversal by DMOG in the central nervous system, providing potential therapy targets and a molecular basis for manganism or Parkinson disease.

## Results

### DMOG impaired MnCl2-induced neurotoxicity in human SH-SY5Y cells

**Figure 1:**
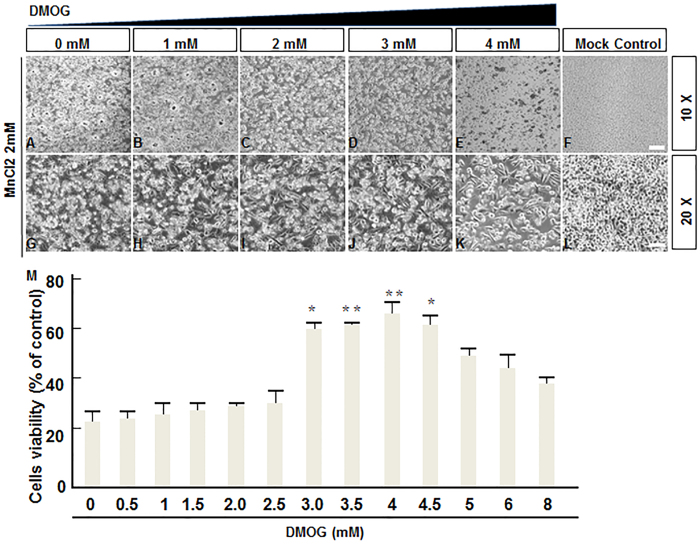
DMOG impaired MnCl2-induced neurotoxicity in human SH-SY5Y cells. (**A–E**) Representative micrographs of SH-SY5Y cells after 1 day (24 h) of treatment with MnCl2 (2 mM) and DMOG of different concentrations (**A–E**); Micrographs with higher magnification (20X) are also shown (**G–K**). Untreated cells as controls (**F,L**). Scale bars, 50 μm (**A–F**); 100 μm (**G–L**). (**M**) Antagonistic effects of DMOG on MnCl2-induced cytotoxicity in human SH-SY5Y cells. Cell viability was determined as described in the Materials and Methods and expressed as a percentage of the control cells (Treated with vehicle control (0.2% water)). Results are means ± SEM of three separate experiments performed in triplicate. *p < 0.05; **p < 0.005 compared to the timed-matched vehicle control (0).

To assess whether DMOG could be used to treat neurodegenerative disorders caused by manganese overexposure, the human neuroblastoma cell line SH-SY5Y, which can serve as an *in vitro* cell model of dopaminergic neurons, was used. Our results showed 24 hours after 2 mM MnCl2 administration, the viability of SH-SY5Y cells decreased to 50% compared with controls (Data not shown). To determine the potential attenuation effects of DMOG with respect to MnCl2-induced cytotoxicity, we pre-treated the SH-SY5Y cells with various concentrations of DMOG 3 hours before the addition of 2 mM MnCl2 solution. Notably, no obvious differences in cell morphology were observed when low doses of DMOG were administered in concentrations up to and including 3 mM (Fig. 1). When cells were pre-treated with DMOG at concentrations higher than 3 mM, half of the cells exhibited normal morphology (Fig. 1D,E) in comparison with the MnCl2-only treatment group (Fig. 1A) and the mock treatment group (Fig. 1F). Representative micrographs at higher magnifications (20X) are also shown (Fig. 1G–L). Furthermore, we estimated the cell viability at 24 h after the administration of various concentrations of DMOG by using a Cell Counting Kit-8 (CCK-8) (Fig. 1M). Consistent with the changes in cell morphology observed under microscope, we noted that, without DMOG pre-treatment, 2 mM MnCl2 administration decreased cell viability to approximately 20% by 24 h. No obvious effects of DMOG administration were observed in concentrations under 3 mM. Pre-treatment with DMOG of 3 mM to 4.5 mM dramatically increased the viability of SH-SY5Y cells to 60–65%, and the highest live cell percentage was observed at 4 mM of DMOG treatment. We also observed a detectable decrease in cell viability when pre-treated with DMOG at concentrations of 4.5 mM or higher compared with the 4 mM treatment group (Fig. 1M). These results derived from SH-SY5Y cells *in vitro* indicate that when cells are pre-treated with DMOG at appropriate concentrations, the cytotoxicity caused by excessive MnCl2 exposure is greatly antagonized.

### Partial restoration effects of DMOG in neurodegenerative disorder in a manganism mouse model

Given the protective effects of DMOG against MnCl2-induced cytotoxicity in SH-SY5Y cells, we wondered whether this DMOG antagonism would also arise in the central nervous system *in vivo*. A manganism mouse model was generated using MnCl2 subcutaneous injections at a dose of 5 mg/Kg. MnCl2 administration was performed every Monday and Thursday for 4 consecutive weeks, and the same amount of physiological saline was used for mock-treatment controls. The body weight and behavioural capabilities of manganism mice were evaluated every week compared to controls. As expected, consecutive administration of MnCl2 decreased body weights and impaired behavioural mobility significantly (Fig. S1). Four weeks later, mice were sacrificed and substantia nigra sections were prepared. Tyrosine hydroxylase staining showed that MnCl2 treatment decreased the number of TH-positive cells to 20% compared to the control group (Fig. S1).

**Figure 2:**
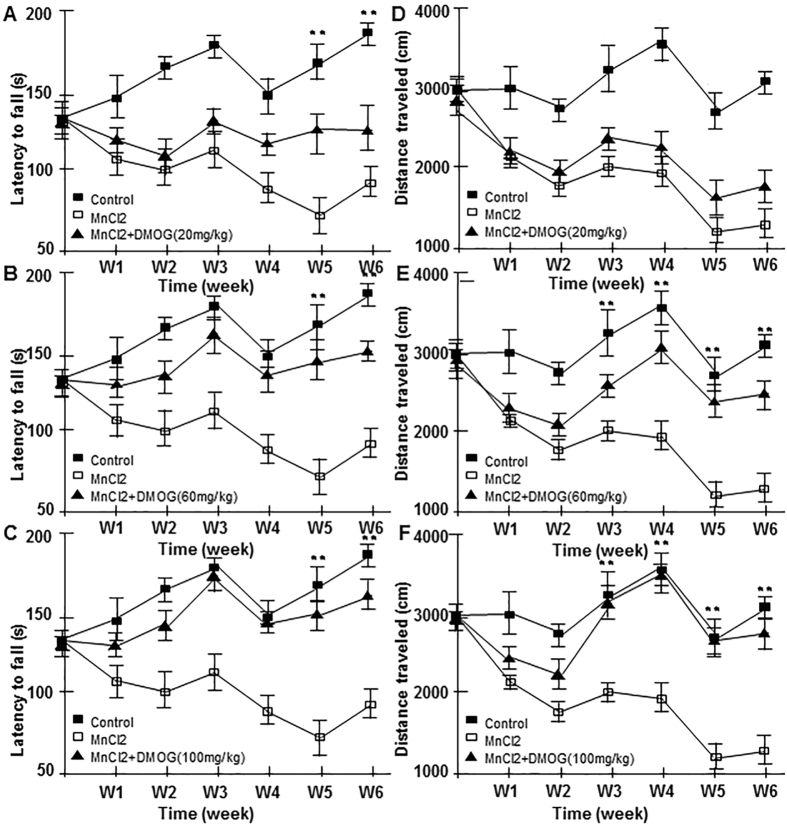
Restorative effects of DMOG on mouse behavioural impairments caused by excessive MnCl2 subcutaneous injection. (**A–C**) Latency to fall in the rotarod test after MnCl2 only or MnCl2 plus various concentrations of DMOG. (**D,E**) Total distance travelled by mice treated with MnCl2 only or MnCl2 plus various concentrations of DMOG. Latency times and distance/length were measured at 6 consecutive time points from week 1 to week 6, respectively. Data are presented as their means ± SEM. **p < 0.01, combination of MnCl2 and DMOG group vs. MnCl2 only group.

We then evaluated the potential antagonistic effects of DMOG using a combined administration of MnCl2 and DMOG. To obtain better comparisons and assessments, we prolonged the treatment time to 6 weeks and 3 unique dosage levels of DMOG (20, 60 and 100 mg/Kg) were used. Injections were performed in separate groups, respectively. Intriguingly, combination injection of MnCl2 and DMOG partially restored the behavioural impairments caused by MnCl2 in a dose-dependent manner (Fig. 2). The rotarod test showed that, even at low doses of DMOG (20 mg/Kg), DMOG administration exhibited a distinguishable restoration effect after only a single injection. Six consecutive combined injections of DMOG (20 mg/Kg) increased the latency from 80 s to 120 s on average (Fig. 2A). In our results, the usage of a medium dose DMOG (60 mg/Kg) brought about a moderate antagonistic effect to the behavioural impairments caused by MnCl2, while high doses of DMOG (100 mg/Kg) almost increased the latency time to normal after 6 weeks of administration (Fig. 2B,C). As with the rotarod test, antagonistic effects of DMOG were also observed in the open field test. Although no obvious effects were observed at week 1, consecutive administration of low or medium doses of DMOG injection still reversed the behavioural impairments compared to the MnCl2-only treatment group. High-dose DMOG (100 mg/Kg) administration increased the travel distance of the mice to nearly the levels of the control group with mock treatment (Fig. 2D–F). These results indicate that DMOG administration antagonizes MnCl2-induced motor deficits in mice.

### DMOG attenuates MnCl2-induced nigrostriatal dopaminergic degeneration in mice

**Figure 3:**
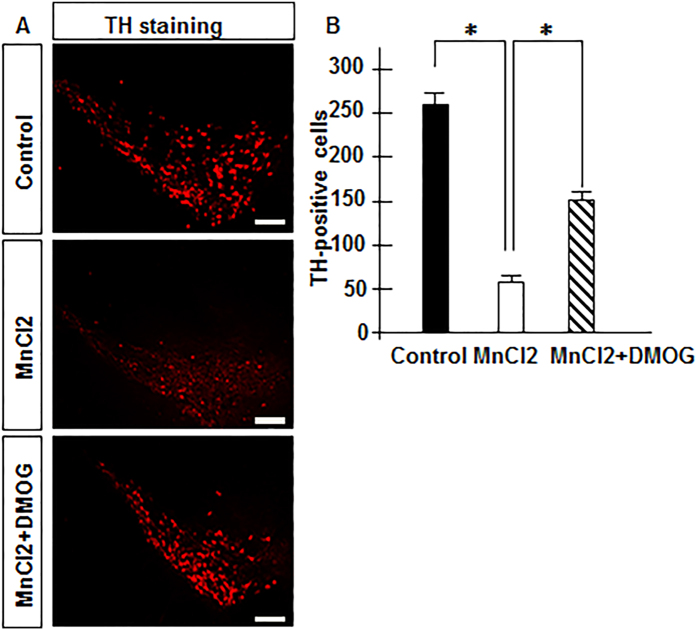
Restorative effects of DMOG on decreased tyrosine hydroxylase positive neurons caused by MnCl2 subcutaneous injection. Representative photomicrographs of immunofluorescence staining for tyrosine hydroxylase (TH) in the substantia nigra pars compacta (SNc) on 30 m-thick coronal sections are shown (**A**). Scale bar, 50 μm. (**B**) Quantification of number of TH positive neurons in SNc is shown. Data are expressed as their means ± SEM from 6 mice per group. Statistical analyses were carried out using one-way or two-way ANOVA. *p < 0.05.

Tyrosine hydroxylase (TH) is a useful marker of dopaminergic central nervous. In our study, we wondered whether DMOG was involved in the pathological processes of manganism in a mouse model. We compared TH expression in the substantia nigra pars compacta (SNc) among groups with different levels of drug administration. After 6 consecutive intraperitoneal injections of DMOG and subsequent behavioural tests, mice were perfused and their TH expression levels were evaluated in the SNc with immunofluorescence. As expected, TH expression was greatly reduced by MnCl2 treatment, but it could be effectively restored by combined DMOG (100 mg/Kg) administration (Fig. 3A). Our estimations of the numbers of TH-positive cells also showed that MnCl2 treatment decreased TH-positive neurons in the SNc, but that this reduction could be greatly restored by combined administration with DMOG (Fig. 3B). The number of TH-positive neurons (approximately 250 per scope) in the control samples was lowered to approximately 50 after MnCl2 exposure, while it increased to 150 on average with a combined DMOG dose. Together, these analyses demonstrate that additional DMOG administration leads to significant antagonism of MnCl2-induced dopaminergic degeneration in mice.

### Genome-wide DNA methylation analyses of DNA

**Figure 4:**
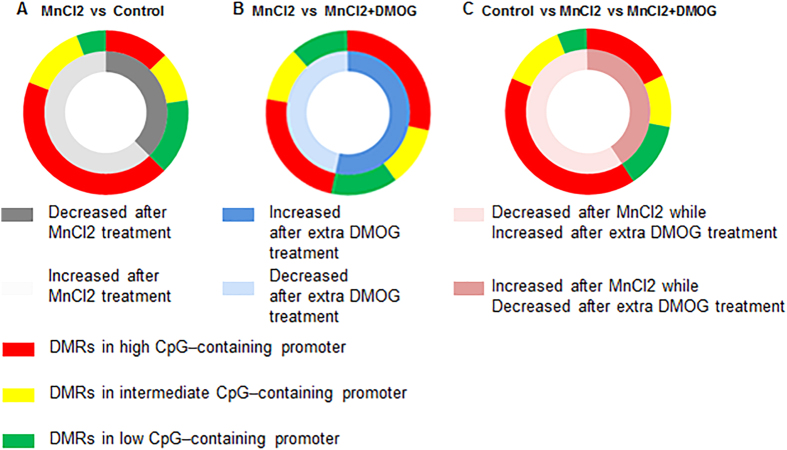
Differentially methylated regions (DMRs) classified into various genetic features, including high, intermediate and low CpG–containing promoters, are analysed between or among the different treatment groups. (**A**) MnCl2 group vs. Control group; (**B**) MnCl2 group vs MnCl2 + DMOG group; (**C**) Control group vs. MnCl2 group vs. MnCl2 + DMOG group. In triple-group comparisons (**C**), only DMRs with restored methylation after combined DMOG treatment are analysed.

Previous results have shown that manganese overexposure causes changes in the methylation status of specific genes during neurodegenerative disorders. Our observations of the antagonistic effects of DMOG during MnCl2-induced dopaminergic degeneration hint at the possibility that such DNA methylation changes are also involved in the protective effects of DMOG. We conducted a genome-wide methylation DNA analysis using microarray hybridization to detect potential methylation changes in our mouse model when challenged with various drugs. Among the two samples of MnCl2 and the control treatment groups, changes in methylation status were revealed in the promoter regions of 913 genes in the substantia nigra of mice after overdose of MnCl2 treatment. Compared with normal controls, the promoter methylation of 571 genes was inhibited by MnCl2 treatment. Various location types and different methylation regions (DMRs) were also analysed. 399 DMRs were found in high CpG–containing promoters (HCP) while 119 and 53 DMRs were distributed in intermediate (ICP) and low CpG–containing promoters (LCP), respectively. Furthermore, MnCl2 administration upregulated the methylation levels of 342 gene promoters, of which, 117, 88 and 137 DMRs were distributed across high, intermediate and low CpG–containing promoters, respectively (Fig. 4A). We also compared methylation changes among the MnCl2 and combination-drug treatment groups. Our results showed that additional DMOG (100 mg/Kg) administration participated in methylation changes in 755 genes in the substantia nigra of mice, in which the promoter methylation of 351 genes was decreased while 404 regions were increased after 6-weeks of consecutive intraperitoneal injection with combined DMOG compared to with MnCl2-only treatment. Different types of DMR distribution in gene promoter regions between the MnCl2 and combination-drug treatment groups are also listed in Fig. 4B. Moreover, the methylation of 226 gene promoters was found to be associated with MnCl2, but this methylation could be restored with combined DMOG treatment (Fig. 4B). Of these, 133 DMRs were distributed across high CpG–containing promoters, while 51 and 42 DMRs, respectively, were found in intermediate and low CpG–containing promoters. Statistics for all genes with changed methylation states and the genetic features of all corresponding DMRs are listed in Table 1.

### Genes with restored methylation after combined DMOG administration in the mouse substantia nigra

The above results demonstrate that global changes in DNA methylation are associated not only with the processes of MnCl2-induced neurodegenerative disorders but also with the antagonistic effects of DMOG. Across all genes with changed methylation, we follow closely those genes with MnCl2-induced but DMOG-restored DNA methylation change in promoter regions, since the reverse change status of DNA methylation represent those genes might function in the processes of cytotoxicity caused by excessive manganese exposure and antagonistic effects of DMOG. Using microarray hybridization with DNA fragments derived from MeDIP enrichment, 226 genes were found to meet the criteria and are listed in File S1 with their annotations. Partial interesting genes were clustered according to their possible functions. We noted that quite a few genes were clustered into three categories as follows: mitochondrial-function associated, cell-cycle and DNA-damage response associated and ion-transportation associated. These clusterings are listed in Table 2, and the genes in same category suggest their potential similar significance in the progress of DMOG antagonism.

## Discussion

The purpose of our current work was to reveal the potential antagonistic effects of the HIF-1 prolyl-hydroxylase inhibitor DMOG in MnCl2-induced neurodegenerative disorders and to determine whether these effects were associated with the DNA methylation of related genes. Our current results suggest that DMOG could partially attenuate the behavioural deficits in mice and the decrease of dopaminergic neurons caused by excessive MnCl2 injection *in vivo*. Meanwhile, our research based on the neuroblastoma cell line SH-SY5Y also showed a similar protective function for DMOG in MnCl2-induced cytotoxicity *in vitro*. In our study, we detected a genome-wide change in DNA methylation during the onset of MnCl2-induced neurodegenerative disorders and a corresponding protective effect derived from DMOG antagonism. A manganism mouse model based on consecutive MnCl2 injections was used in our study because the neurological symptoms of manganism resemble several clinical disorders collectively described as extrapyramidal motor system dysfunction disorders, in particular, IPD and dystonia^26^. Our mouse model also represents common neurodegenerative disorders caused by other industrial chemicals and pesticides. Our results demonstrated the successful generation of manganism in male C57BL/6 mice with consecutive subcutaneous injections for 4 weeks, and the mice exhibited typical symptoms including behavioural deficits and the loss of dopaminergic neurons. Furthermore, for the first time we verified that combined administration of DMOG can partially antagonize MnCl2- induced neurodegenerative disorders in a dose-dependent manner. Human immortalized SH-SY5Y cells from a well-characterized neuroblastoma cell line were chosen for this study because, similar to the central nervous system *in vivo*, these cells show dopamine-β-hydroxylase activity and also express dopaminergic markers. As such, they have been used to study Parkinson’s disease^27^. In our results, pre-treatment with DMOG at concentrations of 3 mM or higher efficiently attenuated MnCl2-induced cytotoxicity. In our cell viability test (Fig. 1M), when cells were pre-treated with DMOG at concentrations of 4.5 mM or higher, cell viability decreased in a dose-dependent manner compared with the 3 mM DMOG group. We speculate this might be due to toxicity caused by the overdose of DMOG itself.

We attribute the antagonistic effects of combined DMOG administration to the changed expression levels of interrelated genes. Our previous results showed that HIF-1α functions as a transcriptional activator for the expression of ATP13A2^23^. In SH-SY5Y cells, real-time PCR showed that MnCl2 decreases but DMOG increases the transcription of ATP13A2. This downregulation of ATP13A2 expression was reversed when cells were challenged with a combination of MnCl2 and DMOG, and a similar regulation was also observed in another ion-transportation associated gene, ATP7B (Fig. S2). The transcriptional regulation of ATP13A2 and ATP7B with MnCl2 and DMOG suggests to us that changes in the expression of related genes are associated with MnCl2-induced neurodegenerative disorders and their antagonism by DMOG. Recently, emerging results have shown that DNA methylation plays an important role in various major neurodegenerative diseases, including Alzheimer’s disease (AD)^28^, Parkinson’s disease (PD)^29^, Huntington’s disease (HD)^30^, and amyotrophic lateral sclerosis (ALS)^31^. Meanwhile, methylation changes in a series of important functional genes have been found to be correlated with neurodegeneration in multiple diseases. Considering these previous findings, we wondered whether changes in DNA methylation were also associated with the antagonistic effects of DMOG. Our genome-wide methylation analysis, based upon microarray hybridization, provides a new understanding of the molecular mechanisms involved in the progress of the DMOG antagonism. In the masses of genes which exhibit changed methylation during manganism or DMOG antagonism, we chose to pay more attention to genes whose methylation was changed by MnCl2 treatment but for which variation could be reversed by combined DMOG administration. Interestingly, a total of 226 genes meeting our criteria were revealed, and a functional clustering was performed. Across all 226 genes, we noted that part of them are correlated to mitochondrial function, cell cycle and DNA damage response or ion transportation (Table 2).

It has been reported that mitochondrial dysfunction is a key contributing factor in PD pathogenesis^32^,^33^,^34^. The mutation or reduced expression of several genes including PINK1, FBXO7, DJ-1, LRRK2, etc. has been found to be associated with mitochondrial dysfunction in neurodegenerative diseases^35^. In our current study, we also found that several mitochondrial-associated genes, including Mars2, Mrpl34, Mterfd1, Pigy and Mrpl42, exhibited changes in DNA methylation when treated with MnCl2 and that these changes could be reversed by combined DMOG administration in the mouse substantia nigra. In addition to the significance of mitochondrial functioning in the intact central nervous system, the loss of genomic maintenance and imperfect DNA repair have also been reported to be prevalent in neurodegenerative disorders. Accumulating evidence shows that the DNA damage response plays an important role in preventing neuropathology^36^,^37^. For instance, oxidative damage to neuronal cells may be an important component of neurodegeneration, as suggested by reports that base excision repair (BER) is important in preventing neurodegeneration^38^. In the functional genes associated with ion transportation, we are most interested in ATP7B. ATP7B is a member of the P-type cation transport ATPase family, and it encodes a protein with several membrane-spanning domains, an ATPase consensus sequence, a hinge domain, a phosphorylation site, and at least 2 putative copper-binding sites. Mutations in this gene have been associated with Wilson’s disease^39^,^40^. Interestingly, patients with Wilson’s disease caused by ATP7B mutation usually also show Parkinson’s like symptoms, such as cogwheel rigidity, bradykinesia or slowed movements and a lack of balance. In our results, we found a dramatic increase in ATP7b expression after DMOG treatment, which was further confirmed with real-time PCR (Fig. S2). Interestingly, the expression of a homologue gene of ATP7b, ATP13a2, was also activated upon DMOG treatment in SH-SY5Y cells. ATP13a2 is reported to protect cells from Mn iron-induced cell death in mammalian cell lines and in primary rat neuronal cultures^41^,^42^. Similarly, it would be quite intriguing to determine whether ATP7b plays a role in protecting against the neurotoxicity caused by manganese in further studies.

Taken together, our study represents the first time that the potent antagonism of DMOG against MnCl2-induced neurodegenerative disorders has been revealed not only *in vivo* but also *in vitro* using a mouse model and the neuroblastoma cell line SH-SY5Y. Furthermore, we are convinced that global methylation changes are associated with this manganese toxicity and DMOG antagonism. Further research will focus on the detailed mechanisms through which alterations in methylation regulate special gene expression and how the changed gene expression participates in MnCl2-induced cytotoxicity and DMOG-induced antagonism in the central nervous system. Taken together, our novel findings provide not only a possible therapy for neurodegenerative diseases but also underlying molecular mechanisms for the generation of neurodegenerative diseases and their corresponding clinical therapy.

## Materials and Methods

### Animals

This study was performed in strict accordance with the recommendations in the Guide for the Care and Use of Laboratory Animals of Central South University. The protocol was approved by the Central South University Animal Care and Use Committee. Experimental mice (Male C57BL/6) were purchased from SLAC Experimental Animal Facility (Shanghai, China). Mice were housed on a 12 h light–dark cycle with free access to food and water. For the manganism mouse model, 7-week old male C57BL/6 mice were housed for 1 week before experiments. Each group, containing at least 5 mice, was assigned randomly. MnCl2 solution (5 mg MnCl2 per 1 Kg body weight) was injected at 9 a.m. every Monday and Thursday for 6 consecutive weeks. For the DMOG antagonism groups, MnCl2 (5 mg/Kg) combined with DMOG (20, 60 or 100 mg/Kg) was injected as above. The same amount of saline was injected as a mock-treatment control.

### Behavioural testing

Motor and non-motor functions were evaluated using a series of behavioural tests including open field locomotor activity and the rotarod test as previous^43^. In order to minimize the possible effects of previous test history, multiple tests were not carried out on the same day. Mice were transported to the behavioural room 1 h prior to tests. All behavioural experiments were performed and the data were analysed blinded with respect to the treatment group.

### Rotarod test

To assess overall motor abilities including coordination, balance and motor learning capabilities when challenged with MnCl2 or the combination of MnCl2 and DMOG, mice were tested at 6 time points over consecutive weeks. An automated accelerating rotarod apparatus DXP-3 was purchased from the Shanghai Institute of Materia Medica of the Chinese Academy of Sciences, and the speed of rod was set at 20 rpm. Each trial lasted 5 min, with a 10 min inter-trial interval. The latency to fall (second) from the rod was recorded. The results are expressed as the average time of 3 trials.

### Open field test

General spontaneous locomotor activity and anxiety-like behaviours in mice were assessed with the open field paradigm with automated video tracking software. Briefly, mice were placed in the centre of the apparatus (square arena, 50 cm × 50 cm × 50 cm) and were allowed to freely explore for 5 min. Their movements were detected with the Open Field Activity System (Hongyi Company, Wuhan, China). The total distance travelled, distance/time travelled in the central area and other parameters were recorded using infrared light-beam breaks. Mice with no movement were excluded from the analysis within 5 min.

### Brain tissue preparation, histology and immunohistochemistry

After consecutive drug treatment and corresponding behavioural testing, mice were euthanized with 10% chloral hydrate, followed by exsanguination to harvest brain tissues. All brain tissues were further micro-dissected to harvest the substantia nigra. Partial tissues were flash-frozen in liquid nitrogen and stored at −80 °C until genome DNA preparation for further MeDIP and methylation microarray analysis. Immunohistochemistry was performed as previously described. Briefly, mice were perfused with 0.1 M PBS (pH 7.4) and fixed with 4% paraformaldehyde in 0.1 M PBS (pH 7.4). The fixed brains were removed and post-fixed overnight in PFA at 4 °C. Gradient dehydration was conducted by using 20% and 30% sucrose dissolved in PBS. Frozen sections (30 μm thickness) of substantia nigra were cut with Leica cryostat and stored at −80 °C for further use. After blocking in PBS containing 5% BSA and 0.3% triton X-100 for 1 h, sections were incubated with primary antibody overnight at 4 °C, followed by incubation at room temperature with appropriate secondary antibodies and developed with DAB. Two-three sections per brain were detected to quantify the density of TH-positive striatal fibres. The images were captured and analysed using the NIS-Elements AR microscope software and the ImageJ software (NIH).

### SH-SY5Y cell cultures, drug treatment and cell viability analyses

Immortalized human neuroblastoma cells from the cell line SH-SY5Y were obtained from ATCC (Manassas, US) and cultured in Dulbecco’s Modified Eagle’s Medium (DMEM, Invitrogen) supplemented with 10% foetal bovine serum (FBS) and 1% penicillin-streptomycin. Cells were maintained at 37 °C in a humidified atmosphere of 5% CO2 in an incubator. Cells were subjected to MnCl2 or DMOG treatment when their confluency reached 70%. Cells were pre-treated with DMOG in the indicated concentrations 3 hours before an extra 2 mM MnCl2 was added. For non-treatment controls, same amount of DMSO dissolved in solution was used. Cell viability was measured with the Dojindo CCK-8 kit (Rockville, MD, USA) as the technical manual.

### MeDIP and Methylation microarray analyses

MeDIP and Methylation microarray analyses followed a previous protocol^44^. Briefly, genomic DNA were extracted and sonicated to random size fragments at approximately 200–1000 bp. Immunoprecipitation of the methylated DNA was performed using BiomagTM magnetic beads coupled with mouse monoclonal antibodies against 5-methylcytidine. The immunoprecipitated DNA was eluted and purified by phenol chloroform extraction and ethanol precipitation. The total input and immunoprecipitated DNA were labelled with Cy3- and Cy5-labeled random 9-mers, respectively, and hybridized on ArrayStar Mouse RefSeq Promoter Arrays, which are multiplex slides with 4 identical arrays per slide. Each array contains 22,327 well-characterized RefSeq promoter regions (from about −1,300 bp to +500 bp of the TSSs), in total covered by ~180,000 probes. Scanning was performed using an Agilent Scanner G2505C.

### Statistical analyses

Statistical analyses were performed using SPSS statistical software (version 16.0). Data are expressed as means ± SEM. Data were analysed by one-way or two-way ANOVA with treatments as factors.

## Additional Information

**How to cite this article**: Yang, N. *et al*. Genome-wide analysis of DNA methylation during antagonism of DMOG to MnCl2-induced cytotoxicity in the mouse substantia nigra. *Sci. Rep*. **6**, 28933; doi: 10.1038/srep28933 (2016).

## Supplementary Material

Supplementary Figure S1

Supplementary Figure S2

## Figures and Tables

**Table 1:** Quantitative statistics of all genes with changed methylation and various distribution types.

Type	Total Number	HCP	ICP	LCP
MnCl2 < Control	571	399	119	53
MnCl2 > Control	342	117	88	137
MnCl2 + DMOG < MnCl2	351	184	81	86
MnCl2 + DMOG > MnCl2	404	216	89	99
MnCl2 < Control &MnCl2 + DMOG > MnCl2	133	92	28	13
MnCl2 > Control &MnCl2 + DMOG < MnCl2	93	41	23	29

**Table 2:** Genes with restored methylation by extra DMOG treatment in mouse substantia nigra.

Gene	Promoter Type	+MnCl2	+DMOG	Strand	Accession	Protein ID	Protein Name
Mitochondrial function associated							
Mars2	HCP	Down	Up	+	NM_175439	NP_780648	methionyl-tRNA synthetase, mitochondrial
Mrpl34	ICP	Down	Up	+	NM_053162	NP_444392	39S ribosomal protein L34, mitochondrial
Mterfd1	ICP	Down	Up	−	NM_025547	NP_079823	mTERF domain-containing protein 1, mitochondrial
Pigy	HCP	Down	Up	−	NM_025574	NP_079850	protein preY, mitochondrial precursor
Mrpl42	ICP	Up	Down	−	NM_026065	NP_080341	39S ribosomal protein L42, mitochondrial
Cell cycle and DNA damage response associated							
Rad9b	HCP	Down	Up	−	NM_144912	NP_659161	cell cycle checkpoint control protein RAD9B
Neil1	LCP	Down	Up	−	NM_028347	NP_082623	endonuclease 8-like 1
Mms19	HCP	Down	Up	+	NM_028152	NP_082428	MMS19 nucleotide excision repair protein
Ercc6l	HCP	Down	Up	−	NM_146235	NP_666347	DNA excision repair protein ERCC-6-like
Trp53i13	HCP	Up	Down	−	NM_001024920	NP_001020091	tumor protein p53-inducible protein 13
Ion transportation associated							
Abca3	HCP	Up	Down	+	NM_001039581	NP_001034670	ATP-binding cassette sub-family A member 3
Atp7b	HCP	Up	Down	−	NM_007511	NP_031537	copper-transporting ATPase 2
