# Supplementary material for: Genome-wide analysis of DNA methylation during antagonism of DMOG to MnCl2-induced cytotoxicity in the mouse substantia nigra
Source: Sci Rep. 2016 Jul 6;6:28933. doi: 10.1038/srep28933 (PMC4933877; doi:10.1038/srep28933)
Supplement: Supplementary Figure S1 [file srep28933-s1.pdf]

Genome-wide analysis of DNA methylation during antagonism of DMOG to MnCl2-induced cytotoxicity in the mouse substantia nigra

Nannan Yang[a #], Yang Wei[a #], Tan Wang[a], Jifeng Guo[a,b,c,d], Qiying Sun[a], Yacen Hu[a], Xinxiang Yan[a,b,d], Xiongwei Zhu[e], Beisha Tang[a,b,c,d], Qian Xu[a]*

[a] Department of Neurology, Xiangya Hospital, Central South University, Changsha, 410008 Hunan, People’s Republic of China.

[b] State Key Laboratory of Medical Genetics, Changsha, 410008 Hunan, People’s Republic of China.

[c] Key Laboratory of Hunan Province in Neurodegenerative Disorders, Central South University, Changsha, 410008 Hunan, People’s Republic of China.

[d] Neurodegenerative Disorders Research Centre, Central South University, Changsha, 410008 Hunan, People’s Republic of China.

[e] Institute of Pathology, Case Western Reserve University, Cleveland, OH 44106. USA.

*Correspondence to: Dr. Qian Xu, Department of Neurology, Xiangya Hospital, Central South University, Changsha, Hunan 410008, People’s Republic of China.

E-mail: xyxuqian2015@163.com

#These authors contributed equally to this work.

Figure S1

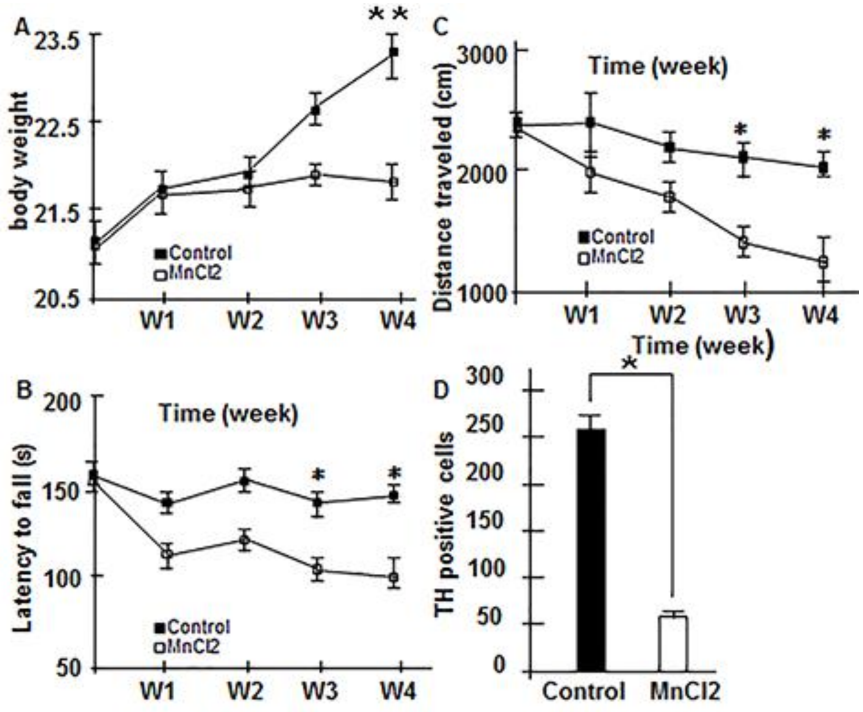

Figure S1. Typical symptoms of manganism in a mouse model caused by consecutive MnCl2 subcutaneous injection. 5 mg/Kg MnCl2 solution was subcutaneously injected into 7-week old mice consecutively on each Monday for 4 weeks. Body weights and motor abilities of experimental and control mice were measured each Thursday. Results from both the experimental and control groups were measured each Thursday (A). Behavioural tests showed excessive MnCl2 treatment impaired the latency to fall of mice by accelerating the rotarod test (B) and shortening the distance travelled in the open field test (C) compared to the mock-treatment control. After 4-week injection of MnCl2, frozen sections of mouse substantia nigra were prepared, and immunofluorescence of tyrosine hydroxylase showed consecutive MnCl2 administration reduced the numbers of TH-positive neurons (D).
