# Supplementary material for: Genome-wide analysis of DNA methylation during antagonism of DMOG to MnCl2-induced cytotoxicity in the mouse substantia nigra
Source: Sci Rep. 2016 Jul 6;6:28933. doi: 10.1038/srep28933 (PMC4933877; doi:10.1038/srep28933)
Supplement: Supplementary Figure S2 [file srep28933-s2.pdf]

Genome-wide analysis of DNA methylation during antagonism of DMOG to MnCl2-induced cytotoxicity in the mouse substantia nigra

Nannan Yang[a #], Yang Wei[a #], Tan Wang[a], Jifeng Guo[a,b,c,d] , Qiying Sun[a], Yacen Hu[a], Xinxiang Yan[a,b,d], Xiongwei Zhu[e], Beisha Tang[a,b,c,d], Qian Xu[a]*

[a] Department of Neurology, Xiangya Hospital, Central South University, Changsha, 410008 Hunan, People's Republic of China.

[b] State Key Laboratory of Medical Genetics, Changsha, 410008 Hunan, People's Republic of China.

[c] Key Laboratory of Hunan Province in Neurodegenerative Disorders, Central South University, Changsha, 410008 Hunan, People's Republic of China.

[d] Neurodegenerative Disorders Research Centre, Central South University, Changsha, 410008 Hunan, People's Republic of China.

[e] Institute of Pathology, Case Western Reserve University, Cleveland, OH 44106. USA.

*Correspondence to: Dr. Qian Xu, Department of Neurology, Xiangya Hospital, Central South University, Changsha, Hunan 410008, People's Republic of China.

E-mail: xyxuqian2015@163.com

#These authors contributed equally to this work.

Figure S2

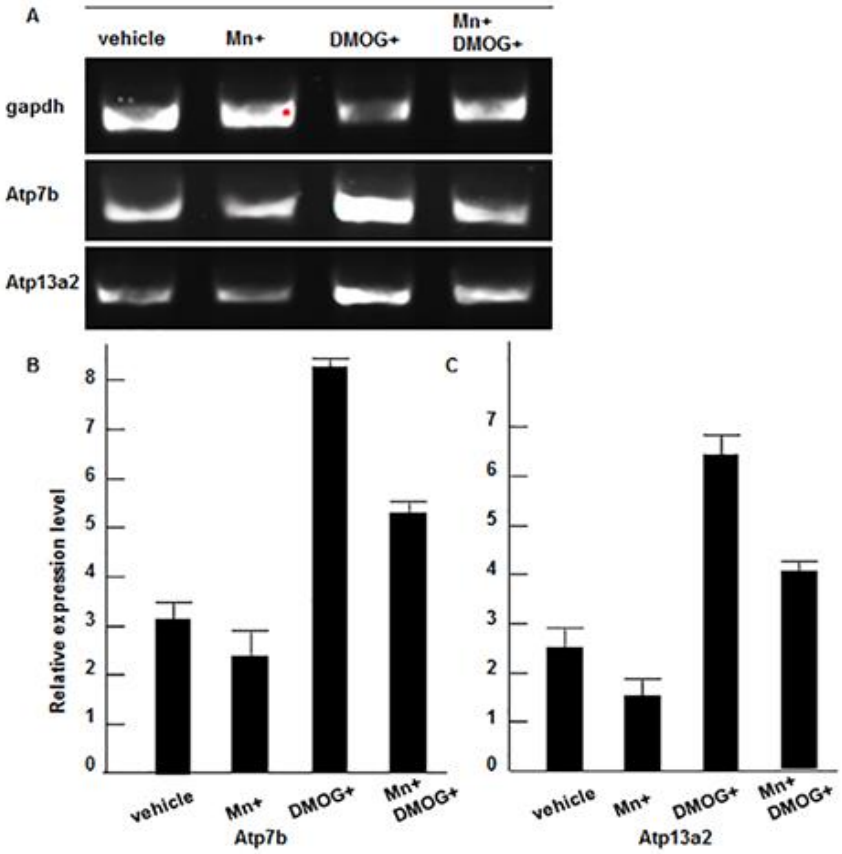

Figure S2. Transcriptional regulation of Atp7b and Atp13a2 genes by MnCl2 and DMOG administration in SH-SY5Y cells. SH-SY5Y cells were treated with MnCl2 (2 mM) or DMOG (3 mM) for 24 hours as indicated, respectively. For the MnCl2 plus DMOG treatment group, cells were pretreated with DMOG (3 mM) for 24 h then treated with MnCl2 for an extra 24 hrs. Semi-quantitative (A) and real-time PCR (B, C) for each gene are shown as figures.
